# Prescribing patterns and costs associated with erectile dysfunction drugs in England: a time trend analysis

**DOI:** 10.3399/bjgpopen20X101145

**Published:** 2021-02-03

**Authors:** Colette Bell, Muhammad Abdul Hadi, Saval Khanal, Vibhu Paudyal

**Affiliations:** 1 Institute of Clinical Sciences, College of Medical and Dental Sciences, University of Birmingham, Birmingham, UK; 2 Behavioural Science Group, Warwick Business School, University of Warwick, Coventry, UK

**Keywords:** erectile dysfunction, deprivation, PDE-5I, prescribing practices, sexual dysfunction, sildenafil citrate, primary health care

## Abstract

**Background:**

Sildenafil and tadalafil are recommended firstline treatments for erectile dysfunction (ED). Sildenafil was legally reclassified to a ‘pharmacy’ medicine in the UK in 2018.

**Aim:**

To assess the prescribing patterns and costs associated with prescribing of ED drugs in England, and to investigate the link between prescribing and deprivation, regional demography, and legal reclassification.

**Design & setting:**

Analyses were conducted of publicly available government data from various sources pertaining to primary care prescribing and demographics in England.

**Method:**

Prescribing and cost data from January 2009 to November 2019 were extracted and adjusted for inflation, male populations, and regional deprivation.

**Results:**

Between 2009 and 2019 the rate of prescribing, measured as the number of items per 1000 men, increased by 110%. In 2019, the rate of prescribing of ED medicines in the most deprived areas was 21.0% higher than the rate observed in least deprived areas. The northern regions of England approximately had a 50% higher rate of prescribing compared with London. A 1.3% annual increase in the number of prescription items was observed between 2018 and 2019 for Sildenafil, with a 5.8% increase observed from 2017–2018.

**Conclusion:**

The two-fold increase in the rate of primary care prescriptions in the past 10 years suggests that more men are being screened for, or seeking help for, ED. The higher rate of prescribing offers opportunity for monitoring of linked risk factors, such as diabetes mellitus, dyslipidaemia, and vascular disorders, in deprived populations. Reclassification of sildenafil had a modest impact on prescribing practices.

## How this fits in

There is a dearth of research in relation to presentation and management of ED in primary care, particularly in the UK. This study shows that over the past 10 years, there has been more than a two-fold increase in the rate of primary care prescriptions for ED, accounting for the changes in population. The reclassification of sildenafil as a pharmacy medicine in 2018 seemed to have modest impact on the prescribing trend. It is very likely that more men are being assessed for ED or seeking help in primary care in England. In addition, data suggest that prescribing is linked with deprivation. There is an opportunity in primary care for assessment of linked risk factors in men who present with symptoms.

## Introduction

ED, ‘the persistent inability to achieve or maintain an erection that is sufficient to permit satisfactory sexual performance’, can affect the physical, emotional, and psychosocial health of the sufferer.^1^ ED can have both an organic and psychogenic origin, and can be a risk marker for underlying cardiovascular diseases (CVDs) and mental health conditions, which may warrant further evaluation and treatment.^2,3^ Medications, such as antihypertensives, antidepressants, and antipsychotics, can also lead to ED through their mechanisms of action.^2^ A systematic review reported that men with ED have an increased risk of all-cause mortality (odds ratio [OR] = 1.26; 95% confidence interval [CI] = 1.01 to 1.57) and CVD mortality (OR = 1.43; 95% CI = 1.00 to 2.05).^4^

The global prevalence of ED has been estimated to range from 3%–76.5%.^4^ A significant number of patients are known to suffer in isolation and do not seek help. Therefore, prevalence data are likely to be an underestimate.^5^ It is common for men with ED to experience anxiety, depression, relationship difficulties, and a lack of sexual confidence.^2,3^ These factors often lead to poorer quality of life for the sufferer, their partner, and family.

Both pharmacological and non-pharmacological treatment options are available for the treatment of ED. The National Institute for Health and Care Excellence (NICE) in England recommends using phosphodiesterase type-five inhibitors (PDE-5I) as a firstline pharmacological treatment option for men with ED. Four PDE-5Is are currently licensed in the UK and include sildenafil, tadalafil, vardenafil, and avanafil.^5^ However, only sildenafil and tadalafil remain the drugs of choice.

Sildenafil was reclassified in March 2018 by the UK’s Medicines and Healthcare products Regulatory Agency (MHRA) to ‘pharmacy-only’ medicine, which allowed community and online pharmacies to supply the drug to the patients without the need of a prescription.^6^ MHRA make decisions to alter the legal status of medicines in the UK from ‘prescription-only’ to ‘pharmacy’ or ‘general sales list’ categories (Table 1).^7,8^ The decision to reclassify such medicines are primarily based on safety data.^7^ Such reclassifications are often expected to reduce the volume of prescribing and costs from the perspective of the NHS as patients are required to pay the price of the medicines at the point of purchase.

**Table 1.** Legal classification of medicines in the UK

**Prescription-only medicines (POM**)	**Pharmacy (P**)	**General sale list (GSL**)
Available only under a prescription from a medical or a non-medical prescriber such as doctors, dentists, or a healthcare professional with an independent prescribing qualification and authority	Available under the supervision of a pharmacist from a pharmacy registered with the General Pharmaceutical Council (GPhC)	Available in general retail outlets such as supermarkets

A cardiovascular risk assessment by a pharmacist in the community pharmacy is a prerequisite to pharmacy supply of sildenafil.^9^ Such risk assessment is undertaken using a series of questions asked to the patients before a supply can be made. Sildenafil is still available on NHS prescription along with tadalafil, vardenafil, and avanafil.

There is a lack of research in relation to presentation and management of ED in primary care, particularly in the UK. To date, no longitudinal research has looked into the prescribing patterns and costs of PDE-5Is in English primary care settings. Given the association of ED with CVDs, including diabetes, vascular disorders, and mental health conditions,^2,3,10^ understanding any link that exists between deprivation and ED drugs prescribing trend will enable targeted interventions to manage comorbidities and reduce health inequality.

The primary aim of this study was to investigate the trend in quantity and cost of prescribing of sildenafil and tadalafil in primary care settings in England between 2009 and 2019. Specific objectives related to the investigation of: a) whether a prescribing trend of ED drugs is associated with deprivation and demography of various English geographical regions; and b) whether the legal reclassification of sildenafil to ‘pharmacy-only’ medicine has impacted on the trend in quantity and cost of prescribing of ED drugs in England.

## Method

### Study design

A secondary analysis of routinely collected data on the prescribing and dispensing of two ED drugs, sildenafil and tadalafil, within the NHS England primary care settings between January 2009 and November 2019, was conducted. This time period includes switches from patented medicines to generic drugs for both tadalafil and sildenafil, as well as the reclassification of sildenafil to ‘pharmacy-only’ medicine in March 2018.

### Data collection

Prescribing datasets were extracted from NHS Digital sources including OpenPrescribing.net and Prescription Cost Analysis (PCA).^11^ These included data on the number of items prescribed, cost of prescribing, and prescribing patterns within specific clinical commissioning groups (CCGs) for both drugs. Data were adjusted for inflation using the Bank of England inflation calculator based on Office for National Statistics (ONS) composite price index.^12^ Data were also adjusted for male population estimates for each year.^13^ All data were extracted, independently checked for accuracy, and analysed using Microsoft Excel and IBM SPSS Statistics (version 21). Prescription patterns in the 10 most and the 10 least deprived CCGs as per the ONS Index of Multiple Deprivation (IMD) in 2015 (Table 2) were also extracted and analysed to explore the link between prescribing pattern and deprivation. The CCGs are clinically-led autonomous NHS bodies involved in planning and commissioning healthcare services for their locality. The 10 most deprived and the 10 least deprived CCGs covered a population of 1.38 million and 1.30 million, respectively.

**Table 2.** Top 10 most and least deprived CCGs in England according to the Index of Multiple Deprivation

**10 most deprived CCGs** **(relevant commissioning regions**)	**IMD rank**	**10 least deprived CCGs** **(relevant commissioning regions**)	**IMD rank**
NHS Bradford City CCG (North East and Yorkshire)	1	NHS Bracknell and Ascot CCG (South East)	200
NHS North Manchester CCG (North West)	2	NHS North East Hampshire and Farnham CCG (South East)	201
NHS Central Manchester CCG (North West)	3	NHS Windsor, Ascot and Maidenhead CCG (South East)	202
NHS Barking and Dagenham CCG (London)	4	NHS Chiltern CCG (South East)	203
NHS Sandwell and West Birmingham CCG (Midlands)	5	NHS Surrey Health CCG (South East)	204
NHS Blackpool CCG (North West)	6	NHS Horsham and Mid Sussex CCG (South East)	205
NHS City and Hackney CCG (London)	7	NHS Guildford and Waverley CCG (South East)	206
NHS Knowsley CCG (North West)	8	NHS Surrey Downs CCG (South East)	207
NHS Tower Hamlets CCG (London)	9	NHS Rushcliffe CCG (Midlands)	208
NHS Liverpool CCG (North West)	10	NHS Wokingham CCG (South East)	209

CCG = clinical commissioning group. IMD = Index of Multiple Deprivation.

## Results

**Figure 1.**
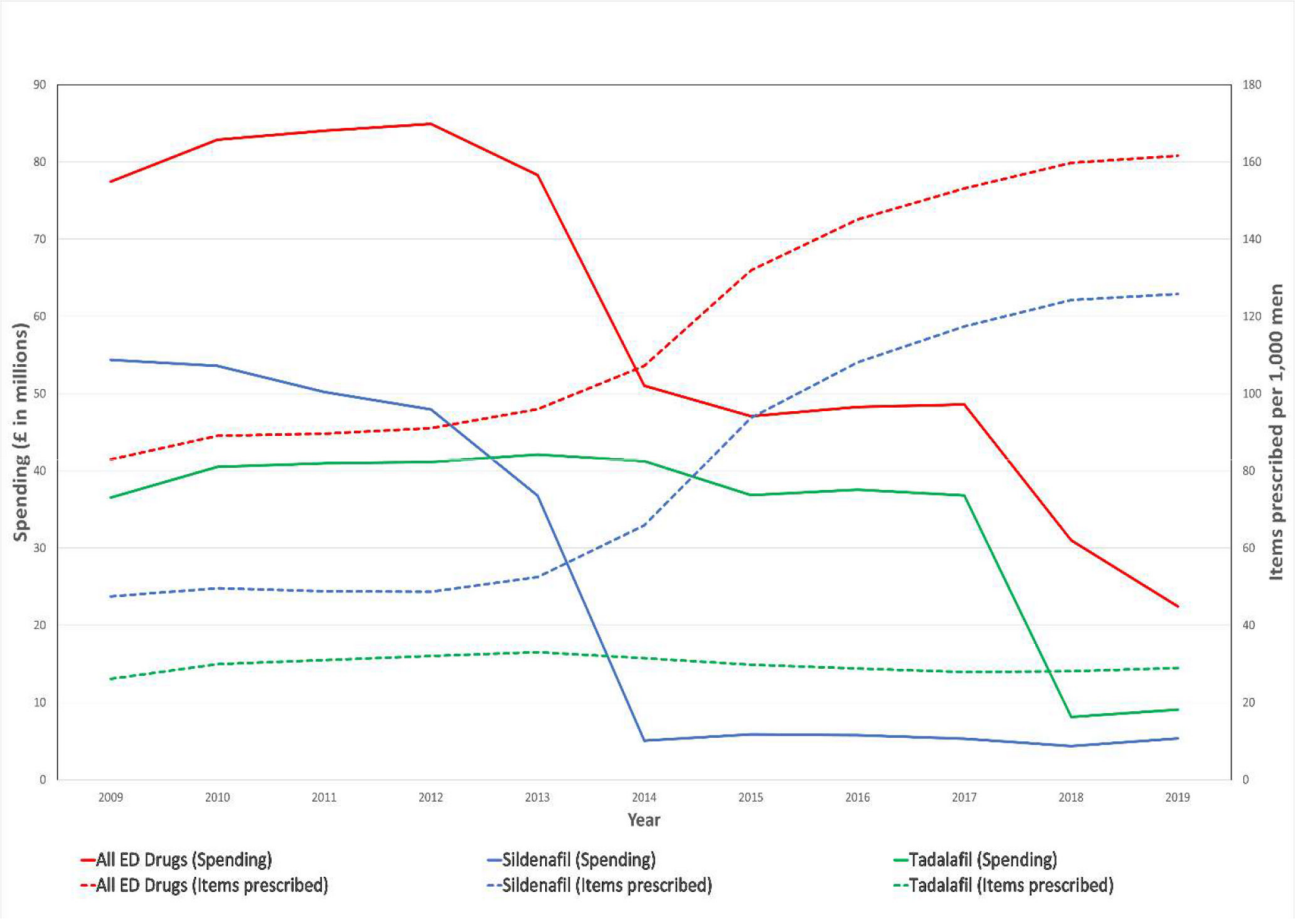
Volume and costs of prescribing for ED medicines from 2009–2019 in English primary care. ED = erectile dysfunction

Number of items prescribed for ED increased by 110% between 2009 and 2019 (Figure 1). The increase in the number of items prescribed was mostly accounted for by sildenafil prescriptions, which increased by 165%. There were 2 145 393 items for ED prescribed in 2009 and 4 505 623 in 2019. The biggest year-on-year increase in the number of prescriptions was seen in 2014–2015, where an increase of 24% was observed.

The total items prescribed for sildenafil was relatively steady from 2009–2013, at around 48 items prescribed a year per 1000 men. The prescribing increased rapidly from 2013–2017. The highest yearly increase occurred from 2014–2015, which showed a 42.1% increase. A 5.8% yearly increase in the rate of prescription was observed between 2017 and 2018. A 0.5% increase in the rate of prescribing between 2018 and 2019 was observed, suggesting modest impact of reclassification on the prescribing practices. The number of items prescribed per 1000 men were 124.2 and 125.8 in 2018 and 2019, respectively.

The spending on primary care prescriptions for ED decreased by 71.0% from £77.4 million in 2009 to £22.4 million in 2019. The spending on sildenafil decreased by 90% and tadalafil decreased by 75%. The biggest decreases were seen after 2014 for sildenafil and 2018 for tadalafil (Figure 1).

### Deprivation and prescribing patterns

The most deprived regions consistently showed higher rates of prescriptions for ED drugs. In 2019 there were 190.4 items prescribed per 1000 men of all ED drugs in the most deprived region. This figure was approximately 21.0% higher compared with the data from the least deprived regions, where a total of 150.5 items were prescribed per 1000 men.

**Figure 2.**
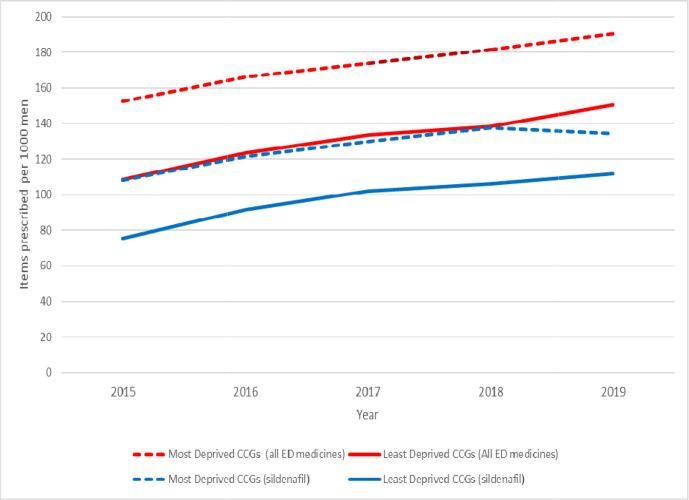
The number of items of ED medicines prescribed annually in the most and least deprived CCGs in England. CCG = clinical commissioning groups. ED = erectile dysfunction

In the most deprived regions, the yearly increases in prescription items were 9.0% in 2016, 4.5% in 2017, and 4.4% and 5.0% in 2018 and 2019, respectively. An overall 24.9% increase in prescription items was observed from 2015–2019. In the least deprived region, the rate of increase in the number of prescriptions was 38.9% during the same period (Figure 2).

### Regional variations in prescribing

**Figure 3.**
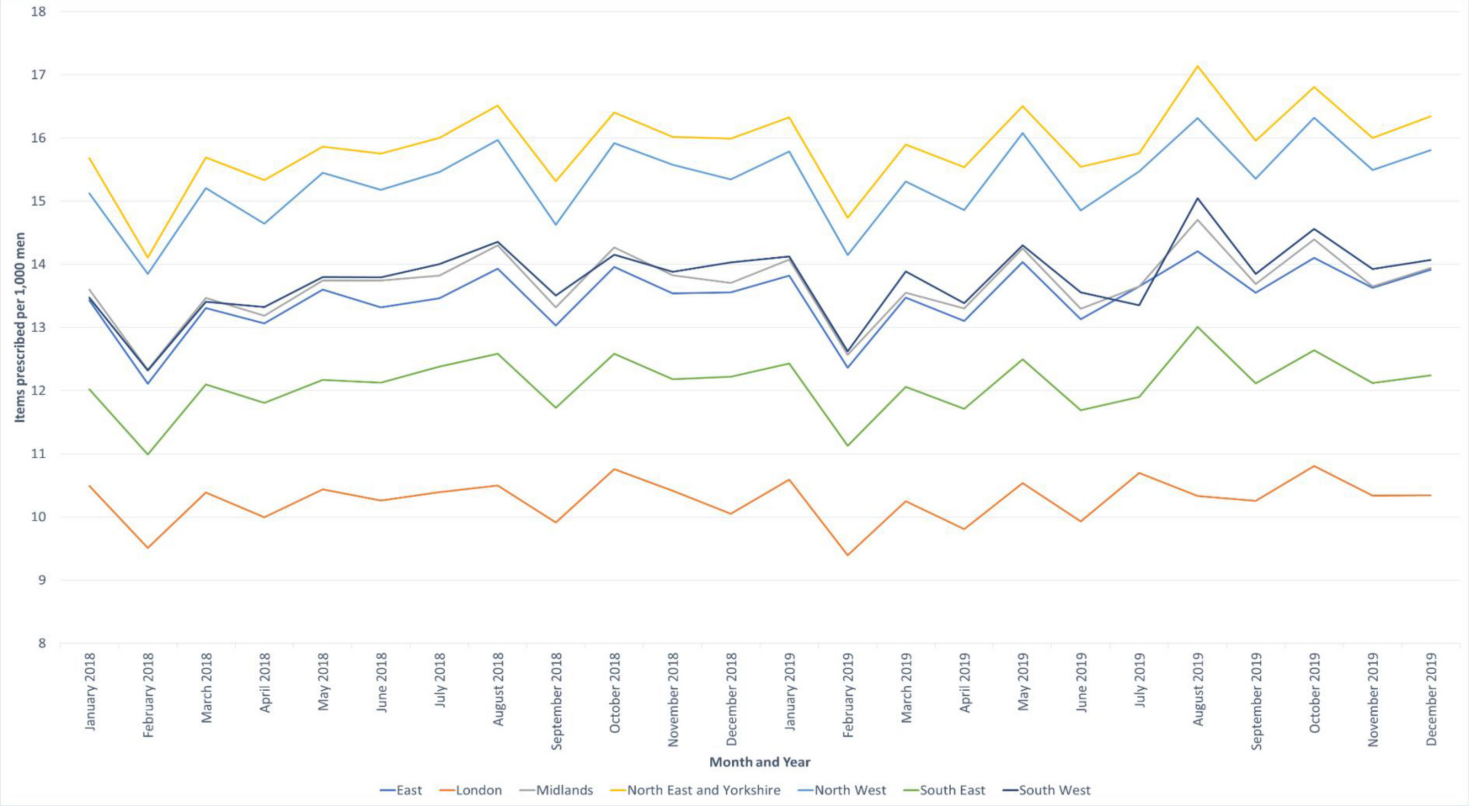
The number of erectile dysfunction medicine items prescribed per month in each NHS commissioning region in 2018 and 2019

A wide variation in the rate of prescribing across the six English regions were observed. The North East and the North West regions had approximately 50% higher rates of prescribing compared with London. Rates of prescribing were 15.9, 15.3, and 10.3 items per 1000 men each month, respectively, in these regions (Figure 3).

## Discussion

### Summary

The aim of this study was to investigate the trend in prescribing of drugs for ED in primary care settings in England and to assess the impact of deprivation, regional demography, and legal reclassification of sildenafil on prescribing practices of ED drugs.

There was a persistent increase in the rate of prescribing for treatment of ED in men between 2009 and 2019 in English primary care. Sildenafil remained the most frequently prescribed medication out of the two drugs of choice. A strong link between deprivation and prescribing trends were observed across all data points. These can be linked to higher prevalence of risk factors of ED, such as cardiovascular and mental health conditions, with deprivation.^14^ A north–south divide was observed in the rate of prescriptions, with higher prescribing rates observed in the northern regions of England compared with London and the southern regions. While some of the regional variations are explained by the differences in deprivation level, London’s younger population compared with the rest of the country^15^ could explain low ED prevalence and the observed low prescribing for ED. In addition, patient behaviours around use of online sources for access to sildenafil may be different across various geographical regions.

Data showed that reclassification of sildenafil to a pharmacy medicine from ‘prescription-only' medicine had modest impact on the trend of prescribing. It could be assumed that the reclassification of sildenafil would lead to a decrease in the number of items prescribed as people would be able to get their medicine directly from the pharmacy instead of needing to go to the doctors and wait for an appointment. It is important to note, however, that the pharmacy version of sildenafil costs approximately £20 for a pack of four tablets (as of April 2020) whereas the generic sildenafil prescriptions from a GP only costs £9 for the same number of tablets. The higher cost of the pharmacy version of the medicine compared with the prescription cost may dissuade patients from using pharmacy for accessing sildenafil. In addition, confidentiality and privacy have been cited as barriers to utilisation of sexual health services from pharmacy.^16^ The lack of long-term monitoring and follow-up in pharmacy settings may also discourage many patients from accessing the pharmacy version of sildenafil.

### Strengths and limitations

This project included data from very large routinely collected datasets covering all primary care prescriptions in England. Deprivation data were based on CCG level and individual practice level variations were not accounted for. This study only included NHS prescription data and private prescriptions were not accounted for. Only two PDE-5Is were included, sildenafil and tadalafil, as the authors' observations suggested that the rest of the PED-5Is accounted for negligible volume of prescribing and, hence, did not impact on the trend analysis. Similarly, community and online pharmacy sales of over-the-counter sildenafil were not included in the trend analysis. Some patients may avoid perceived embarrassment and confidentiality issues in face-to-face clinical consultations in primary care and community pharmacy^16^ by accessing medicines through online sources. Other available treatments for ED, including vacuum-pump treatment and psychosexual therapies, were not included in the study. In addition, prevalence data of ED over time were considered for this study. The segmented regression for different policy changes (for example, sildenafil patent expiry in June 2013, tadalafil patent expiry in November 2017, and reclassification of sildenafil from prescription medicine to pharmacy medicine in March 2018) could not be performed owing to: a) absence of monthly prescribing and spending data (access was restricted to annual level for historical data prior to 2015); and b) inadequate time-points before and after different policy changes needed to conduct segmented regression analysis.^17^

### Comparison with existing literature

There is a lack of literature on the prescribing and management of ED in primary care. Previous studies have reported that many men do not seek help with sexual problems to avoid perceived embarrassment for themselves and their doctors.^18,19^ The observed increasing trend in the rate of prescriptions may have been contributed by various factors. These include inclusion of ED assessment for men with diabetes in the Quality and Outcomes Framework (QOF) since 2013–2014.^20^ The QOF required opportunistic screening, advice and assessment of contributory factors, and treatment options for ED in patients with diabetes in primary care. In addition, media, including social media, may have contributed to increased knowledge and awareness of the condition among members of the general public given the licensing of newer PDE-5Is in recent years.

The study shows that the introduction of generic versions of both sildenafil in 2013 and tadalafil in 2017 reduced overall spending on drugs used for ED. The expiry of the patent on sildenafil in June 2013 allowed the NHS to purchase generic sildenafil from other pharmaceutical companies for a competitive price, subsequently reducing the price per item from £31.31 in 2012 to £2.53 in 2014.^11^ This also allowed sildenafil to be removed from the Selected List Scheme, which restricted prescriptions of certain drugs in May 2014, allowing more men access to the medicine on the NHS, which potentially increased the number of items prescribed.^21^ The patent on tadalafil also expired in November 2017, leading to a 49% decrease in cost per item from £49.40 in 2017 to £25.12 in 2018.

### Implications for research and practice

The data suggest that more men are seeking help or being assessed for ED in primary care. The increased presentations and, hence, prescribing for EDs allows further opportunities to screen for the associated risk factors for ED. These include: CVDs such as hypertension, coronary artery disease, peripheral vasculopathy; endocrine disorders such as diabetes, metabolic syndrome, and hyperthyroidisim; as well as assessment of psychogenic risk factors and medication-induced ED.^2^ Prescribers should be aware of the wider organic, psychogenic nature and linked risk factors of ED including diabetes, vascular disorders, and mental health conditions. The cause of ED needs to be established before treatment can commence and patients should be referred to specialist clinics where appropriate. Guidelines recommend that a physical examination, including genitourinary, endocrine, vascular, and neurological systems, is undertaken. This can reveal any unsuspected diagnoses, such as Peyronie’s disease, pre-malignant or malignant genital lesions, prostatic enlargement or irregularity or nodularity, or signs and symptoms suggesting hypogonadism.^2,3^ It is, however, unclear how often patients are screened for the red-flag symptoms. Long-term safety outcomes of PDE-5Is are not understood. Prescribers should, therefore, reassess the continuous use of these drugs on a periodic basis.^22,23^

Reclassification to allow ‘pharmacy-only’ supply of drugs to free-up GP time and resources have faced successes and barriers in the past.^24–26^ For example, reclassification of drugs such as chloramphenicol eye products, mild steroids, and antifungal products have been positively received by patients, GPs, and pharmacists. However, reclassified pharmacy version of simvastatin was not adopted in practice to the same extent.^24^ Research on community pharmacist and patient perspectives on the need, supply, and aspects of risk assessments and referrals are warranted. GPs and patient experiences of access to sildenafil from pharmacy also need to be further explored.

Results of this study suggest that more men are being screened for, or seeking help with, ED. The two-fold observed increase in the population adjusted rate of prescriptions over the past 10 years is less likely to be accounted for by other factors. Promotion of sildenafil by pharmaceutical industries since being made available over the counter for pharmacy sales could have raised awareness of ED among the general public. Higher rate of prescribing in deprived regions offers opportunity for monitoring of linked health conditions such as CVD and diabetes. Reclassification of sildenafil to pharmacy medicine had a modest impact on prescribing practices.
